# Investigating the relationship between predictability and imbalance in minimisation: a simulation study

**DOI:** 10.1186/1745-6215-14-86

**Published:** 2013-03-27

**Authors:** Gladys C McPherson, Marion K Campbell, Diana R Elbourne

**Affiliations:** 1 Health Services Research Unit, University of Aberdeen, Health Sciences Building, 3rd Floor Foresterhill, Aberdeen, AB25 2ZD, UK; 2 Medical Statistics Unit, London School of Hygiene and Tropical Medicine, London, WC1E 7HT, UK

**Keywords:** Randomisation, Minimisation, Allocation imbalance, Allocation predictability

## Abstract

**Background:**

The use of restricted randomisation methods such as minimisation is increasing. This paper investigates under what conditions it is preferable to use restricted randomisation in order to achieve balance between treatment groups at baseline with regard to important prognostic factors and whether trialists should be concerned that minimisation may be considered deterministic.

**Methods:**

Using minimisation as the randomisation algorithm, treatment allocation was simulated for hypothetical patients entering a theoretical study having values for prognostic factors randomly assigned with a stipulated probability. The number of times the allocation could have been determined with certainty and the imbalances which might occur following randomisation using minimisation were examined.

**Results:**

Overall treatment balance is relatively unaffected by reducing the probability of allocation to optimal treatment group (*P*) but within-variable balance can be affected by any *P* <1. This effect is magnified by increased numbers of prognostic variables, the number of categories within them and the prevalence of these categories within the study population.

**Conclusions:**

In general, for smaller trials, probability of treatment allocation to the treatment group with fewer numbers requires a larger value *P* to keep treatment and variable groups balanced. For larger trials probability of allocation values from *P* = 0.5 to *P* = 0.8 can be used while still maintaining balance. For one prognostic variable there is no significant benefit in terms of predictability in reducing the value of *P*. However, for more than one prognostic variable, significant reduction in levels of predictability can be achieved with the appropriate choice of *P* for the given trial design.

## Background

The main aim of randomisation in randomised clinical trials (RCTs) is to reduce the risk of selection bias at trial entry. A secondary aim is to generate groups that are comparable in terms of key prognostic factors. Clinical trials often enrol sufficiently small numbers that clinically relevant imbalances in prognostic factors between groups may occur by chance, particularly if simple random allocation, for example, a coin toss, is used. In anything but the largest trials there can be imbalances in the distribution of prognostic variables between treatment groups [1]. These differences can weaken the power of the trial and make it hard to distinguish between real and spurious treatment effects.

Toorawara *et al*. [2] wrote that treatment balance may be ‘desirable in a range of scenarios including small trials, interim analyses, early termination, analysis of subgroups or where the credibility of an unbalanced trial may be problematic, e.g. in the case of a small treatment effect.’ Altman and Bland [3] also agree that balance is especially desirable for trials with strong prognostic factors and modest treatment effects. Within-centre imbalance may be considered more problematic than imbalance within other prognostic factors for practical reasons. Resources for a particular treatment may be limited within centres, for example, limited intensive care facilities available for the study, or limited number of surgeons trained in a particular procedure. The Committee for Proprietary Medicinal Products (CPMP) [4] encourages balancing at the centre level, and dynamic allocation techniques can be used to do this. This has led researchers to develop restricted randomisation methods to ensure balance of prognostic variables across treatment groups at baseline. An example of this is the minimisation method, which has been labelled the ‘platinum standard’ of randomisation [5], where for each centeracteristic of interest, the total number of patients within each treatment group is calculated, and the next allocation is assigned to minimise the difference between the groups. However, care must be taken to ensure proper execution of these methods, as errors can be costly. A programming error in the randomisation schedule of the COMET trial [6] led to a severe imbalance in distribution between treatment groups for two prognostic factors, age and ethnicity. The trial team sought advice from the funding body and independent clinical trials experts and re-recruitment of the entire study sample was recommended.

Taves realised that the next assignment could usually be predicted if the exact system used in the minimisation procedure was known [7]. With knowledge of the current group totals the next allocation could be predicted in nearly 70% of cases. Pocock and Simon argued that the probability of assignment to the optimal treatment (that is, the allocation that would minimise the difference between treatment groups) according to the minimisation algorithm should be set at some value less than one to ensure that the next assignment cannot be predicted with certainty [8]. They compared allocation systems where the probability (*P*) of receiving the optimal treatment was reduced from 1.0 to 0.75, and found that this increased the risk of treatment imbalance compared with *P* = 1.0. However, they and others accept that in many trials, particularly multi-centre trials, it is feasible to set this *P* at 1.0 [8,9]. The reasons for this are that the randomisation will usually be managed remotely perhaps by an independent service and the individual centre data will not easily be disentangled from the complete dataset.

To reduce the probability of guessing the treatment allocation when the number in one group is less than the other(s) we can assign a treatment using a *P*-value less than 1.0. It is unclear, however, what effect this would have on the balance between treatment arms and within the levels of any prognostic factor [10].

Against this background the use of restricted randomisation methods, such as minimisation, is increasing. This paper investigates under what conditions it is preferable to use restricted randomisation in order to achieve balance between treatment groups at baseline with regard to important prognostic factors, and whether trialists should be concerned that minimisation may be considered deterministic.

## Methods

Using minimisation as the randomisation algorithm, hypothetical patients entering a theoretical study were assigned values for the prognostic factors by random assignment with varying stipulated probabilities. Treatment allocations of these hypothetical patients were simulated and the imbalances that might occur following randomisation using minimisation were examined.

The following factors were varied in a series of 1,000 simulations: size of trial (N); number of prognostic variables, the number of categories within these variables and the distribution of the population within these categories (called the prevalence within these categories); and the probability of allocation to optimal treatment (*P*). The probability of allocation *P* was varied between *P* = 0.5 (the case of simple randomisation) and *P* = 1 (fully deterministic). These were the values also selected by Hills *et al*. [1] and Brown *et al*. [11] for their simulations. Starting with the simplest case of two treatments, simulations with varying values of *P* were performed, first with one binary variable where patients entering the trial had equal likelihood of presenting with the centeracteristic of either category within the prognostic variable (that is, there was equal prevalence within the population). Then simulations were performed where the prevalence within this variable was not equal within the population, to see if this had an effect on predictability and/or treatment imbalance. Next, the effect of increasing numbers of prognostic variables was explored by increasing these to two, three and four variables. The simulated patient centeracteristics were checked to ensure the simulations had been correctly encoded. Similar sets of simulations were then performed for 3 and 4 treatments to investigate the effect of increasing the number of treatment groups.

Simulations were performed using a Microsoft Access database and programming in VBA (Visual Basic for Applications). The desired centeracteristic variables of N patients were randomly generated with selected probability using inbuilt computer functions for random number generation. These simulated patients were randomised using the minimisation algorithm with the specified probability of optimal treatment allocation. The program recorded whether simple randomisation had been applied (that is, when there was a tie between treatment groups with the smallest number of allocations to date), whether the random twist had been applied (see below for definition), or if the allocation was deterministic (could have been predicted with certainty). The number of times the allocation could have been determined with certainty was calculated as the predictability (see below for definition). The treatment imbalance within prognostic factors and for the trial overall was calculated (see below for definition of imbalance).

### Definition of predictability

The number of times the allocation could have been determined correctly was calculated as the predictability. This assumes that the guesser knows that treatment allocation is using the method of minimisation and knows the exact algorithm for this, knows the centeracteristics and treatment allocations of all patients randomised to date, and is trying to correctly identify the next allocation. For example, using probability of allocation *P* = 1.0, if there is one treatment arm with the smallest number of participants then the allocation would be deterministic and could be predicted. If there was more than one treatment arm with the smallest number of participants then simple randomisation would be used and the treatment would not be predictable (although the user could guess correctly approximately 50% of the time in the two-treatment case).

This definition of predictability differs from the one used by Hills [1] and Brown [11] where they used various guessing strategies based on: a) the previous allocation only; b) the previous allocation to a particular clinician, and c) the previous five allocations to a particular clinician. The method presented in this paper assumes knowledge of all previous allocations for a given clinician or centre, and could therefore represent the case where the randomisation algorithm employs minimisation by one or more factors and stratification by centre. Predictability is measured by knowledge of the algorithm and the centeracteristics of the presenting patients and no guessing of allocation is included. The scenarios where variables have increased numbers of factor levels can be extended to show the effect if centre is incorporated as a minimisation variable.

With *P* <1.0, if the allocation was to the treatment that would have been assigned had *P* been equal to 1.0, then the allocation was again deemed to have been predictable. If the allocation was to the alternative treatment, the random twist (see below) had been applied and the allocation was not predictable.

### Definition of random twist

Random twist occurs when a probability of allocation *P* <1.0 is used to determine treatment allocation in the event of only one treatment group having the least number of patients (in the case of a tie between two or more treatment groups, simple randomisation is used). Using *P* = 1.0 then the treatment allocation is defined here to be predictable; but if *P* <1 is used and the allocation does not go to the expected treatment (the deterministic allocation), the random twist is said to have been applied and the allocation could not have been correctly predicted.

#### Example of minimisation with varying values of P

Assume the first 20 patients entered into a hypothetical trial have the profile shown in Table 1.

**Table 1:** Profile of the first 20 patients entered into a trial

**Prognostic factors**		**Group A**	**Group B**
Gender	Male	3	2
	Female	7	8
Age band, years	<80	6	9
	≥80	4	1
Type of fracture	Proximal femur	0	2
	Distal forearm	4	4
	Clinical vertebral	0	0
	Other	6	4
Time since fracture	0 to 3 months	5	7
	>3 months	5	3

Results are presented as numbers of patients.

If the 21st patient has the factors female, age <80 years, proximal femur, 0 to 3 months:

Total in group A with the same centeracteristics 7 + 6 + 0 + 5 = 18

Total in group B with the same centeracteristics 8 + 9 + 2 + 7 = 27

With *P* = 1.0: the 21st patient is allocated to group A because 18 is <27. The allocation is said to be deterministic.

With *P* = 0.8: the 21st patient is allocated to group A with probability 0.8. The 21st patient is allocated to group B with probability 0.2. If the patient is allocated to group A then the allocation is said to be deterministic. If the patient is allocated to group B then the random twist has been applied. The allocation is truly randomised and could not have been predicted with certainty.

### Definition of imbalance

For N ≥50, imbalance was defined as at least 10% chance of at least 5% absolute imbalance between treatment groups within any of the prognostic factors, or overall treatment imbalance in the trial (that is, at least 100 of the 1,000 simulations resulted in at least 5% absolute imbalance across the groups in the percentage of people with a certain prognostic factor).

For very small values of N (<50), a 5% absolute imbalance represents very small differences in actual numbers, so this value has to be amended for very small trials. When calculating imbalance, the values in Table 2 were used.

**Table 2:** Cutoff for imbalance in small trials

**Size of trial (number of participants)**	**Cutoff for imbalance**
10	40% (7:3)
20	20% (12:8)
30	10% (17:13)
40	10% (22:18)
≥50	5% (27:23)

Cutoff is given in % (numbers in each of the 2 groups in a 2-treatment trial).

## Results

### Observations in the two-treatment case

#### One prognostic variable

In the two-treatment case with one binary variable, equal prevalence in each category and probability of assignment *P* = 1.0, the number of observed ties was 50% and simple randomisation was used in these cases. The percentage of deterministic allocations was 50% when *P* = 1, dropping by 2.0 to 5.0% for 10 ≤ N ≤50; by 1.0 to 1.7% for 60 ≤ N ≤100; and by only 0.1 to 0.5% for N >100 as the value of *P* was decreased to 0.7, and before imbalance was observed. Therefore, for N >50 the number of times the random twist was used, as *P* was decreased, was almost totally offset by the drop in the number of ties giving no overall decrease in the likelihood of predictability with decreasing values of *P*, but at a potential cost in terms of treatment imbalance for N <90. In these cases, treatment imbalance both within prognostic variables and between treatment arms was evident for *P* = 0.7. For N ≥90, good balance was achieved with *P*-values dropping to 0.7 but there was no significant benefit in the decrease in predictability.

When the prevalence in the single binary variable was unequal the observations for the decrease in predictability with changing probability of assignment (*P*) were similar to those for a binary variable with equal prevalence but there was more likelihood of imbalance within prognostic variables at *P* = 0.8 for N <80 and for imbalance at *P* = 0.7 for N up to 100. Overall balance across treatment arms was no different, with unequal prevalence with both scenarios showing imbalance at *P* = 0.7 for N <90; *P* = 0.6 for N = 90 or 100, and at *P* = 0.5 for N ≥200.

In the two-treatment case with one three-category variable the number of ties and predictions with changing probability of assignment was almost the same as with a binary variable (for N = 100 the number of ties was 1% higher and the number of predictions was 1% lower). However, there was a greater chance of imbalance being observed with decreasing values of *P*, especially for smaller trials. Imbalance at both the variable and treatment level was consistently shown for N <90 at *P* = 0.8 and for N ≤100 at *P* = 0.7.

For simulations with one four-category variable the observations for the decrease in predictability with changing probability of assignment (*P*) was almost the same as with a binary variable. Within-variable imbalance was evident for N up to 100 and *P* = 0.8, and there was a greater chance of overall treatment imbalance for N up to 50 with *P* = 0.9 and for N up to 100 with *P* = 0.8.

So, for the simulations with one prognostic variable we conclude that increasing the number of categories within the variable (or selecting categories within the variables that result in unequal distribution of patients within these categories) increase the likelihood of treatment imbalance within categories and between treatment arms overall. However these factors do not significantly affect the predictability of allocation with a given probability of allocation value (*P*).

#### Two prognostic variables

With two binary variables, both with equal prevalence and probability of allocation *P* = 1.0, the number of observed ties was approximately 28% and simple randomisation was used in these cases. The percentage of deterministic allocations was 72% when *P* = 1.0, dropping to around 60% when *P* = 0.7, before imbalance was starting to be observed. Therefore, in this case (unlike the case of only one prognostic factor), there was a benefit of a 12% drop in the number of predictions as the level of *P* was reduced from 1.0 to 0.7. However for N = 100 and *P* = 0.7 there was imbalance at the level of the prognostic variables, so for N ≤100 a value of *P* higher than 0.7 would be recommended. For N <90 there was imbalance observed between treatment arms at *P* = 0.7.

With two binary variables of unequal prevalence the number of ties and predictions was almost the same as two variables with equal prevalence. However, different prevalence within prognostic factors again had an effect on imbalance. For N = 100, there was observed imbalance within prognostic variables when *P* = 0.8, which again demonstrated the effect of prevalence on imbalance. Therefore, although there was a gain in terms of the number of deterministic allocations as the probability of allocation *P* was decreased, this came at the expense of treatment balance across prognostic variables. As above, overall treatment balance was only compromised at *P* = 0.7 for N <90.

In the two-treatment case with two three-category variables with equal prevalence and *P* = 1.0, the number of observed ties was 30% and simple randomisation was used in these cases. The percentage of deterministic allocations was 70% when *P* = 1.0, dropping to 60% when *P* = 0.7. This was almost the same as with two binary variables but there was greater likelihood of imbalance being observed with *P* <1.0 for small trials. Within-variable imbalance was observed with *P* = 0.9 for N = 100 and overall treatment imbalance at *P* = 0.8 for N <100.

For two variables, one binary and one with three categories, both with equal prevalence, for N = 100 there was imbalance within variables at *P* = 0.8 but not at *P* = 0.9 (similar to the simulation with one three-category variable). For two variables, both with four categories, for N = 100 there was imbalance observed within variables at *P* = 0.9 and between treatment arms at *P* = 0.7. For N = 200 there was imbalance within variables at *P* = 0.7.

#### Three prognostic variables

In the two-treatment case with three binary prognostic variables with equal prevalence and *P* = 1.0, the number of observed ties was around 20% and simple randomisation was used in these cases. The percentage of deterministic allocations was approximately 80% when *P* = 1, dropping to around 63% when *P* = 0.7, when imbalance was starting to be observed. Therefore, in this case there was a 17% drop in the number of predictions as the level of *P* is reduced from 1.0 to 0.7. However, when N = 100, there was a potential for imbalance within the variables at *P* = 0.7 and across treatment arms at *P* = 0.7 for N up to 80.

For three binary prognostic variables with different prevalence there was observed imbalance within variables for N up to 100 at *P* = 0.8 and for N = 200 at *P* = 0.7. Again, there was imbalance across treatment arms at *P* = 0.7 for N up to 80.

#### Four prognostic variables

In the two-treatment case with four binary variables all with equal prevalence and *P* = 1.0, the number of observed ties was approximately 15% and simple randomisation was used in these cases. The percentage of deterministic allocations was around 85% when *P* = 1.0, dropping to around 65% when *P* = 0.7, when imbalance was observed. Therefore, in this case, there was a 20% drop in the number of predictions as the level of *P* was reduced from 1.0 to 0.7. However, there was a potential for imbalance when N = 100 and *P* = 0.8; and at N = 200 with *P* = 0.7. Again, there was imbalance across treatment arms at *P* = 0.7 for N up to 80.

For four binary prognostic variables with different prevalence there was observed imbalance within variables for N up to 100 at *P* = 0.9 and for N = 200 at *P* = 0.7. Again, there was imbalance across treatment arms at *P* = 0.7 for N up to 80.

#### Summary of two-treatment case

The size of the trial, number of prognostic variables, number of categories within these and the prevalence of the categories in the study population all affected the point at which imbalance began to be observed with decreasing values of *P*. The number of prognostic variables determined the frequencies of ties and deterministic allocations but the likelihood of imbalance was dependent also on the number of categories within these variables and the prevalence of these centeracteristics within the study population. The higher the number of categories in the prognostic variables and the more uneven the distribution of the centeracteristics in the study population the greater was the chance of observing imbalance with probability of allocation *P* <1.0.

With two treatments and one prognostic variable, there was very little decrease in predictability as probability of allocation *P* was decreased, typically <1%. As the *P-*value was decreased, the number of random twists increased but very often this increase was reflected more in a decrease in the number of simple randomisations taking place (when treatment groups are equal) rather than in a decrease in the number of possible predictions. In this case there appeared to be little advantage to be gained from using a *P* -value less than 1.0.

With two variables predictability can be reduced by 10 to 12% for N >100 when *P* = 0.7 to 0.8, but some scenarios require *P* = 0.9 or *P* = 1.0 to avoid imbalance (Table 3). With three variables predictability can be reduced by up to 17% and with four variables up to 20%, but again some scenarios require *P* = 0.9 or *P* = 1.0 to avoid imbalance (Table 3).

**Table 3:** Summary of observations from the two-treatment case

	**Predictability at *P *= 1.0, %**	**N**^**a**^	**Recommended *P-*value**	**Predictability at this *P-*value, %**	**Reduction in predictability, %**	***P*-value at which imbalance occurs**
**One variable**						
Two categories	50.0	<90	0.8^b^	45.0 to 48.8	1.2 to 5.0	0.7
		90 to <200	0.7^b^	49.0	1.0	0.6
		≥200	0.7^b^	49.5 to 49.9	<1.0	0.5
Two categories - unequal prevalence		< 80	0.9^b^	45.0 to 48.8	1.4 to 5.0	0.8
		80 to <200	0.8^b^	49.0	<1.0	0.7
		≥200	0.7^b^	49.0 to 49.9	<1.0	0.5
Three categories		<90	0.9^b^	45.0 to 48.8	1.2 to 5.0	0.8
		90 to <200	0.8^b^	49.0	1.0 to 1.1	0.7
		200 and <300	0.7^b^	49.0	1.0	0.6
		≥300	0.7^b^	49.3 to 49.6	<1.0	0.5
Four categories		≤100	0.9^b^	40.0 to 49.0	1.0 to 5.0	0.8
		200 and <400	0.7^b^	49.0 to 49.3	0.7 to 1.0	0.6
		≥400	0.7^b^	49.5 to 49.6	<1.0	0.5
**Two variables**						
Both with 2 categories	72.0	100	0.8	63.0	9.0	0.7
		200	0.7	60.0	12.0	0.6
		≥300	0.7	60.0	12.0	0.5
Both with 2 categories - unequal prevalence		<100	1.0	-	-	0.9
		100	0.9	68.0	4.0	0.8
		200	0.7	60.0	12.0	0.6
		≥300	0.7	60.0	12.0	0.5
One with 2 categories, one with 3 categories		100	0.9	68.0	4.0	0.8
		200 to 300	0.7	60.0	12.0	0.6
		≥400	0.7	60.0	12.0	0.5
Both with 3 categories		≤100	1.0	-	-	0.9
		200 to 300	0.7	60.0	12.0	0.6
		≥400	0.7	60.0	12.0	0.5
Both with 4 categories	70.0	≤100	1.0	-	-	0.9
		200	0.8	64.0	6.0	0.7
		300 to 400	0.7	60.0	10.0	0.6
		500	0.7	60.0	10.0	0.5
**Three variables**						
All with 2 categories - equal prevalence	80.0	100	0.8	68.0	12.0	0.7
		200 to 300	0.7	63.0	17.0	0.6
		≥400	0.7	63.0	17.0	0.5
All with 2 categories - unequal prevalence		100	0.9	74.0	6.0	0.8
		200	0.8	69.0	11.0	0.7
		300	0.7	63.0	17.0	0.6
		≥400	0.7	63.0	17.0	0.5
**Four variables**						
All with 2 categories- equal prevalence	85.0	100	0.9	78.0	7.0	0.8
		200	0.8	72.0	13.0	0.7
		300 to 400	0.7	65.0	20.0	0.6
		≥500	0.7	65.0	20.0	0.5
All with 2 categories- unequal prevalence		100	1.0	-	-	0.9
		200	0.8	72.0	13.0	0.7
		300 to 400	0.7	65.0	20.0	0.6
		≥500	0.7	65.0	20.0	0.5

^a^The categories of N are dependent upon the point at which imbalance is observed (the value of probability of assignment *P*). ^b^For one prognostic variable the reduction in predictability is so small as the probability of assignment *P* is reduced that the recommended *P-*value is 1.0.

### Observations in the three-treatment case

#### One prognostic variable

In the three-treatment case, with one binary variable and equal prevalence in each category, and probability of allocation *P* = 1.0, the number of observed ties was 67% and simple randomisation was used in these cases. The percentage of deterministic allocations was 33% when *P* = 1.0, dropping only by 0.5 to 1% for N ≥100 as the value of *P* was decreased to 0.7 and before imbalance began to be observed. Therefore, as in the two-treatment case with one binary variable, the number of times the random twist was used is almost totally offset by the drop in the number of ties, giving no overall decrease in the likelihood of predictability with decreasing values of *P*, but at a potential cost in terms of treatment imbalance. For N = 100, treatment imbalance within prognostic variables was evident for *P* = 0.7. For N >100 good balance was achieved with *P* -values dropping to 0.7 but there was no significant benefit in the decrease in predictability.

When the prevalence in the single binary variable was unequal the observations for the decrease in predictability with changing probability of assignment (*P*) were again negligible but there was more likelihood of imbalance within prognostic variables at *P* = 0.8 for N up to 200. Overall balance across treatment arms was no different with unequal prevalence with both scenarios showing imbalance at *P* = 0.6 for N = 100 and no treatment imbalance for N >100.

In the three-treatment case with one three-category variable the number of ties and predictions with changing probability of assignment was almost the same as with a binary variable (for N = 100 the number of ties was 1% higher and the number of predictions was 1% lower). However, there was a greater chance of observing imbalance with decreasing values of *P*, especially for smaller trials of up to N = 100. Imbalance at the variable level was shown for N = 100 at *P* = 0.9 and between treatment arms at *P* = 0.7. For N >100 good balance was achieved with *P*-values down to 0.6 but there was no significant benefit in terms of reduction in predictability as the *P*-value was decreased.

For simulations with one four-category variable the observations for the decrease in predictability with changing probability of assignment (*P*) was again almost the same as with a binary variable. Within variable imbalance was evident at N = 100 and *P* = 1.0 at the 5% level but not the 10% level and there was a greater chance of overall treatment imbalance for N = 100 with *P* = 0.7. For N = 200 there was a significant likelihood of within variable imbalance at *P* = 0.8 but good balance between treatment arms was achieved with *P* -values down to 0.6.

So, for the simulations with one prognostic variable we conclude that increasing the number of categories within the variable (or selecting categories within the variables that result in unequal distribution of patients within these categories) increased the likelihood of treatment imbalance within categories and between treatment arms overall. However these factors did not significantly affect the predictability of allocation with a given probability value (*P*). Also, reducing the value of *P* associated with treatment allocation did not reduce this level of predictability.

#### Two prognostic variables

With two binary variables both with equal prevalence and *P* = 1.0, the number of observed ties was approximately 44% and simple randomisation was used in these cases. The percentage of deterministic allocations was 56% when *P* = 1.0, dropping to around 48% when *P* = 0.7, before imbalance was starting to be observed. Therefore, in this case (unlike the case of only one prognostic factor), there was evidence of a benefit of an 8% drop in the number of predictions as the level of *P* was reduced from 1.0 to 0.7. However for N = 100 and *P* = 0.8 there was imbalance at the level of the prognostic variables so for N ≤100 a value of *P* higher than 0.8 would be recommended. Imbalance between treatment arms was not reached until *P* = 0.6 for N = 100, *P* = 0.5 for N = 200 and not at all for N >200.

With two binary variables of unequal prevalence the number of ties and predictions was almost the same as two variables with equal prevalence. However, different prevalence within prognostic factors again had an effect on imbalance. For N = 100, there was a potential for imbalance within prognostic variables when *P* = 1.0, which again demonstrated the effect of prevalence on imbalance. Therefore, although there was a gain in terms of a decrease in the number of deterministic allocations as the probability of allocation *P* was decreased, this came at the expense of treatment balance across prognostic variables. As above, overall treatment balance was only compromised at *P* = 0. 6 for N = 100.

#### Three prognostic variables

In the three-treatment case with three binary prognostic variables with equal prevalence and *P* = 1.0, the number of observed ties was around 33% and simple randomisation was used in these cases. The percentage of deterministic allocations was approximately 67% when *P* = 1.0, dropping to around 55% when *P* = 0.7, when imbalance was observed. Therefore, in this case there was a 12% drop in the number of predictions as the level of *P* was reduced from 1.0 to 0.7. However, when N = 100, there was a potential for imbalance within the variables at *P* = 0.9 and imbalance was not observed across treatment arms until *P* = 0.6. For three binary prognostic variables with different prevalence the likelihood of imbalance both within variable and across treatment arms was not significantly different from above.

#### Four prognostic variables

In the three-treatment case with four binary variables all with equal prevalence and *P* = 1.0, the number of observed ties was approximately 26% and simple randomisation was used in these cases. The percentage of deterministic allocations was around 74% when *P* = 1.0, dropping to around 57% when *P* = 0.7, when imbalance began to be observed. Therefore, in this case, there was a 17% drop in the number of predictions as the level of *P* is reduced from 1.0 to 0.7. However, there was a potential for imbalance when N = 100 and *P* = 1.0; and at N = 200 with *P* = 0.7. Again, there was no imbalance across treatment arms until *P* = 0.6.

#### Summary of three-treatment case

With three treatments and one prognostic variable, there was very little decrease in predictability as *P* is decreased, typically <1%. As the *P* -value was decreased, the number of random twists increased but very often this increase was reflected more in a decrease in the number of simple randomisations taking place (when treatment groups are equal) rather than in a decrease in the number of possible predictions. In this case there was little advantage to be gained from using a *P* -value less than 1.0.

With two variables predictability could be reduced by approximately 8% for N >100 when *P* = 0.7, but some scenarios required *P* = 0.9 or *P* = 1.0 to avoid imbalance (Table 4). With three variables predictability could be reduced by up to 12% and with four variables up to 17%, but again some scenarios required *P* = 0.9 or *P* = 1.0 to avoid imbalance (Table 4).

**Table 4:** Summary of observations from the three-treatment case

	**Predictabilityat *P *= 1.0, %**	**N**^**a**^	**Recommended *P*-value**	**Predictability at this *P*-value, %**	**Reduction in predictability, %**	***P*-value at which imbalance occurs**
**One variable**						
Two categories	33.0	100	0.8^b^	32.0	1.0	0.7
		200 to 300	0.7 ^b^	33.0	0	0.5
		≥400	0.7 ^b^	33.0	0	-
Two categories - unequal prevalence		100	1.0	33.0	0	1.0
		200	0.8 ^b^	33.0	0	0.7
		300	0.7 ^b^	33.0	0	0.5
		≥400	0.7 ^b^	33.0	0	-
Three categories		100	1.0	32.0	0	0.9
		200	0.7 ^b^	32.5	<1.0	0.6
		300	0.7 ^b^	33.0	<1.0	0.5
		≥400	0.7 ^b^	33.0	<1.0	-
Four categories		100	1.0	32.0	0	0.9
		200	0.9 ^b^	32.5	<1.0	0.8
		300	0.7 ^b^	32.7	<1.0	0.6
		≥400	0.7 ^b^	32.5	<1.0	0.5
**Two variables**						
Both with 2 categories	56.0	100	0.9	54.0	2.0	0.8
		200	0.7	48.0	8.0	0.6
		300	0.7	48.0	8.0	0.5
		≥400	0.7	48.0	8.0	-
Both with 2 categories - unequal prevalence		100	1.0	56.0	0	1.0
		200	0.9	54.0	2.0	0.8
		300	0.7	49.0	7.0	0.6
		≥400	0.7	48.0	8.0	0.5
**Three variables**						
All with 2 categories - equal prevalence	67.0	100	1.0	67.0	0	0.9
		200 to 300	0.7	55.0	12.0	0.6
		≥400	0.7	56.0	11.0	-
All with 2 categories - unequal prevalence		100	1.0	67.0	0	0.9
		200 to 300	0.7	55.0	12.0	0.6
		≥400	0.7	55.0	12.0	0.5
**Four variables**						
All with 2 categories - equal prevalence	74.0	100	1.0	74.0	0	1.0
		200	0.8	64.0	10.0	0.7
		300	0.7	57.0	17.0	0.6
		≥400	0.7	58.0	16.0	0.5

^a^The categories of N are dependent upon the point at which imbalance is observed (the value of probability of assignment *P*). ^b^For one prognostic variable the reduction in predictability is so small as the probability of assignment *P* is reduced that the recommended *P-*value is 1.0.

### Observations in the four-treatment case

#### One prognostic variable

In the four-treatment case with one binary variable and equal prevalence in each category and probability of allocation *P* = 1.0, the number of observed ties was approximately 76% and simple randomisation was used in these cases. The percentage of deterministic allocations was 24% when *P* = 1.0, and did not decrease at all for N ≥100 as the value of *P* was decreased to 0.6, and before imbalance was observed. Therefore, as in the two- and three-treatment cases with one binary variable, the number of times the random twist was used was almost totally offset by the drop in the number of ties, giving no overall decrease in the likelihood of predictability with decreasing values of *P*, but at a potential cost in terms of treatment imbalance. For N ≥100, treatment imbalance within prognostic variables was evident for *P* -values dropping to 0.6 but there was no significant benefit in the decrease in predictability.

#### Two prognostic variables

With two binary variables, both with equal prevalence and *P* = 1.0, the number of observed ties was approximately 55% and simple randomisation was used in these cases. The percentage of deterministic allocations was 45% when *P* = 1.0, dropping to around 41% when *P* = 0.7, before imbalance was observed for N >100. Therefore, in this case (unlike the case of only one prognostic factor), there was a benefit of a 4% drop in the number of predictions as the level of *P* was reduced from 1.0 to 0.7. However for N = 100 and *P* = 0.8 there was imbalance at the level of the prognostic variables, so for N ≤100 a value of *P* higher than 0.8 would be recommended. Imbalance between treatment arms was not reached until *P* = 0.6 for N = 100 and not at all for N ≥200.

#### Three prognostic variables

In the three-treatment case with three binary prognostic variables with equal prevalence and *P* = 1.0, the number of observed ties was around 42% and simple randomisation was used in these cases. The percentage of deterministic allocations was approximately 58% when *P* = 1.0, dropping to around 49% for N >100 when *P* = 0.7, when imbalance was observed. Therefore, in this case there was a 9% drop in the number of predictions as the level of *P* was reduced from 1.0 to 0.7. However, when N = 100, there was a potential for imbalance within the variables even at *P* = 1.0 and imbalance was not reached across treatment arms until *P* = 0.6.

#### Four prognostic variables

In the four-treatment case with four binary variables all with equal prevalence and *P* = 1.0, the number of observed ties was approximately 33% and simple randomisation was used in these cases. The percentage of deterministic allocations was around 67% when *P* = 1.0, dropping to around 53% for N >100 and *P* = 0.7, when imbalance was observed. Therefore, in this case, there was a 14% drop in the number of predictions as the level of *P* was reduced from 1.0 to 0.7. However, there was a potential for imbalance when N = 100 and *P* = 1.0, and at N = 200 with *P* = 0.7. Again, there was no imbalance evident across treatment arms until *P* = 0.6 (Table 5).

**Table 5:** Summary of observations from the four-treatment case

	**Predictability at *P *= 1.0, %**	**N**^**a**^	**Recommended *P*-value**	**Predictability at this *P*-value, %**	**Reduction in predictability, %**	***P*-value at which imbalance occurs**
**One variable**						
Two categories (equal prevalence)	24 to 25	100	0.7^b^	24	0	0.6
		≥200	0.7 ^b^	25	0	-
**Two variables**						
Both with 2 categories (equal prevalence)	45	100	0.9	44	1	0.8
		200	0.7	41	4	0.6
		≥300	0.7	41	4	-
**Three variables**						
All with 2 categories (equal prevalence)	58	100	1.0	58	0	1.0
		200	0.7	49	9	0.6
		300	0.7	49	9	0.5
		≥400	0.7	49	9	-
**Four variables**						
All with 2 categories (equal prevalence)	67	100	1.0	67	0	1.0
		200	0.8	59	8	0.7
		300	0.7	53	14	0.5
		≥400	0.7	53	14	-

^a^The categories of N are dependent upon the point at which imbalance is observed (the value of probability of assignment *P*). ^b^For one prognostic variable the reduction in predictability is so small as the probability of assignment *P* is reduced that the recommended *P-*value is 1.0.

Table 6 summarises the maximum benefit in terms of decreasing predictability for different treatment and variable combinations. For one prognostic variable there was no great benefit in the drop in predictability values as probability of allocation (*P*) was decreased regardless of the number of treatments or the number of prognostic variables but significant benefits could be achieved when more prognostic variables were incorporated into the algorithm.

**Table 6:** Maximum decrease in predictability for different treatment and variable combinations

	**One variable**	**Two variables**	**Three variables**	**Four variables**
Two treatments	<1%	4 to 12%	6 to 17%	7 to 20%
Three treatments	<1%	2 to 8%	11 to 12%	10 to 17%
Four treatments	<1%	Maximum 4%	Maximum 9%	8 to 14%

## Discussion

A major purported concern with constrained methods of randomisation is that they may increase the predictability of the next treatment allocation and this could lead to selection bias. Chalmers *et al*. [12] reviewed 145 papers published between 1946 and 1981 and found imbalance in at least one prognostic variable in 14.0% of blinded randomisation studies, 26.7% of unblinded studies and 58.1% of non-randomised studies. They attributed the imbalance to bias in treatment and suggested that this was possibly due to investigators being able to predict the next treatment. However, imbalances may occur by chance, without investigator bias. For example, in 2002 the American Academy of Emergency Medicine commissioned an independent panel to reanalyse the data from a 1995 trial (the NINDS trial published in the New England Journal of Medicine) on the basis of imbalance in patient centeracteristics at baseline, which they felt may have invalidated the whole trial [13]. After detailed analysis, the committee concluded that the results were indeed valid. Nevertheless, imbalances can lead to questions about the credibility of results.

It is unclear how often imbalances occur in large late-phase trials but empirical evidence from three multi-centre studies has shown how using simple randomisation could result in serious baseline imbalance at crucial analyses points within small, medium and large trials [14]. Some trialists believe that although restricted randomisation may improve precision and reduce bias in small trials, it would be safer to address any imbalance with statistical corrections [15]. However, others maintain that ‘statistical efficiency is of no comfort to someone whose trial is seriously imbalanced and may be suspected of providing a biased estimate of the treatment effect’ [16]. This viewpoint is also reinforced by CPMP [4] who state that in the case of ‘very strong baseline imbalance, no adjustment may be sufficiently convincing to restore the results.’ In 2005, Senn outlined three misunderstandings about randomisation - ‘randomisation does not guarantee balance, balanced covariates may not be ignored and the possible distribution of unmeasured covariates in a validly randomised trial does not invalidate the probability statements about the effect of the treatment’ [17]. This paper has concentrated, therefore, on the ability of the method of minimisation to balance for known prognostic variables and how best to achieve this without compromising allocation predictability. Of course, this is only applicable to studies where the treatment allocations are not blinded. Taves maintained that if the study cannot be double-blind then some randomisation should be added to the minimisation and suggested that the issue of whether randomisation should always be added to minimisation to prevent selection bias in trials that cannot be double-blinded, is due for re-evaluation [18].

The International Conference on Harmonisation (ICH) E9 guideline [19] states that although unrestricted randomisation is an acceptable approach, dynamic allocation procedures may help to achieve balance across a number of factors. However, they indicate that deterministic dynamic procedures should be avoided and an appropriate element of randomisation should be incorporated for each treatment allocation. A deterministic procedure would allocate to the treatment group with the lowest totals with certainty (that is, *P* = 1), and so the allocation could be guessed if the centeracteristics of all previously randomised patients and their treatment allocations were known. The CPMP [4] regards dynamic allocation as highly controversial, even if deterministic schemes are avoided. They, therefore, advise avoidance of such methods and if they are used they should be justified both clinically and statistically.

Prediction rates increase if the randomisation algorithm incorporates stratification by centre (since this produces a row in the minimisation table for each centre or a list per centre for blocked randomisation). Prediction rates decrease if centre is used as a minimisation factor since each centre does not know the centeracteristics or treatment allocation of participants from the other centres.

The results presented in this paper demonstrate that in general for smaller trials, a larger probability of allocation value, *P*, is needed to keep treatment and variable groups balanced. For larger trials, *P* <0.8 can be used while still maintaining balance but the benefits for decreasing predictability is variable. If interim analysis is planned for a trial of more than two treatments it would be preferable to wait until at least 100 patients had been recruited in order to compare the groups without any complicated statistical methods as the potential for imbalance is high.

For one prognostic variable there was no great benefit in the drop in predictability values as *P* was decreased regardless of the number of treatments or the number of prognostic variables. For two treatments and one prognostic variable simple randomisation is used 50% of the time. Therefore, the random twist can only potentially be applied to the other 50% of randomisations. Even with a lower probability of allocation to the optimal treatment (*P*) this results in very few cases in which the random twist will be applied for small trials.

So, there is no benefit in employing a complicated algorithm to try and reduce predictability when there is only one prognostic variable incorporated into the minimisation algorithm. The recommendation here would be to either use random permuted blocked randomisation or, if using minimisation, use a probability of allocation *P* = 1 and incorporate some other method to reduce predictability, such as a) concealing the prognostic variables and/or categories within these from the recruitment personnel; b) including collection of a variable that is not used for minimisation at the time of randomisation, or c) incorporating centre as a minimisation variable. However, significant reduction in the level of predictability can be achieved when there is more than one prognostic variable with the appropriate choice of *P* (as seen in Table 6).

Generally speaking, overall treatment balance is relatively unaffected by reducing the probability *P* but within-variable balance can be affected by any *P* -value less than 1. This effect is magnified by higher numbers of prognostic variables, the number of categories within them and the prevalence of these categories within the study population.

## Conclusions

Trialists have continued to debate the use of minimisation for treatment allocation in clinical trials [3,16-18] and many still advocate its use. The reasons they give for this are mainly because of resource and organisational issues at a centre level, and to provide baseline balance. Some continue to be concerned about the predictability of the method but recognise that it may be possible to use the simple case with probability of allocation *P* = 1 and use other techniques to control this. Methods that can be used to limit predictability include not giving the exact details of the minimisation algorithm in the protocol (recommended by ICH [19]), adding in other factors to produce noise, alternating between randomisation and minimisation, or incorporating centre as a minimisation variable.

The simulations performed for this paper looked closely at the effect of varying the probability of treatment allocation *P* as a method to reduce the predictability of minimisation, and the effect of this on balance within prognostic variables and treatment groups. In general, for smaller trials, the probability of treatment allocation to the treatment group with fewer numbers requires a larger value *P* in order to keep treatment and variable groups balanced. For larger trials probability values between *P* = 0.5 and *P* = 0.8 can be used while still maintaining balance. For one prognostic variable there is no significant benefit in terms of predictability in reducing the value of *P*. However, for more than one prognostic variable, significant reduction in the level of predictability can be achieved with the appropriate choice of value of *P* for the given trial design.

## Abbreviations

CPMP: Committee for Proprietary Medicinal Products; IHC: International Conference on Harmonisation; RCT: Randomised clinical trial; VBA: Visual Basic for Applications

## Competing interests

All authors declare that they have no competing interests.

## Authors’ contributions

The material presented here formed part of GCM's PhD thesis; MKC and DRE were co-supervisors. GCM carried out the simulations and wrote the first draft of the paper. GCM, MKC and DRE were all involved in the development and refinement of subsequent drafts. GCM is the guarantor for the study. All authors read and approved the final manuscript.

## References

[B1] Hills RK Gray R Wheatley K Balancing treatment allocations by clinician or center in randomized trials allows unacceptable levels of treatment prediction J Evid Base Med 2009 2 196 204 10.1111/j.1756-5391.2009.01023.x 21349013

[B2] Toorawara R Adena M Donovan M Jones S Conlon J Use of simulation to compare the performance of minimization with blocked randomization Pharm Stat 2009 8 264 278 10.1002/pst.346 18756580

[B3] Altman DG Bland JM Treatment allocation by minimisation BMJ 2005 330 843 10.1136/bmj.330.7495.843 15817555 PMC556084

[B4] Committee for Proprietary Medicinal Products (CPMP), The European Agency for the Evaluation of Medicinal Products (EMEA) Points to consider on adjustment for baseline covariates 2003 London: European Medicines Evaluation Agency 1 10

[B5] Treasure T Macrae KD Minimisation: the platinum standard for trials? Randomisation doesn't guarantee similarity of groups; minimisation does BMJ 1998 317 362 363 10.1136/bmj.317.7155.362 9694748 PMC1113668

[B6] COMET Study - Comparative Obstetric Mobile Epidural Trial Study Group UK Effect on low-dose mobile versus traditional epidural techniques on mode of delivery: a randomised controlled trial Lancet 2001 358 19 23 11454372 10.1016/S0140-6736(00)05251-X

[B7] Taves DR Minimisation: A new method of assigning patients to treatment and control groups Clin Pharmacol Ther 1974 15 443 453 4597226 10.1002/cpt1974155443

[B8] Pocock SJ Simon R Sequential treatment assignment with balancing for prognostic factors in the controlled clinical trial Biometrics 1975 31 March 103 115 1100130

[B9] White SJ Freedman LS Allocation of patients to treatment groups in a controlled clinical study Br J Cancer 1978 37 849 857 10.1038/bjc.1978.124 350254 PMC2009630

[B10] Hills R Gray R Wheatley K High probability of guessing next treatment allocation with minimisation by clinician [abstract] Control Clin Trials 2003 24 Suppl 3S 70S

[B11] Brown S Thorpe H Hawkins H Brown J Minimization - reducing predictability for multi-centre trials whilst retaining balance within centre Stat Med 2005 24 3715 3727 10.1002/sim.2391 16320287

[B12] Chalmers TC Celano P Sacks HS Bias in treatment assignment in controlled trials N Engl J Med 1983 309 1358 1361 10.1056/NEJM198312013092204 6633598

[B13] Ingall T O’Fallon W Asplund K Findings from the reanalysis of the NINDS tissue plasminogen activator for acute ischemic stroke treatment trial Stroke 2004 35 2418 24 10.1161/01.STR.0000140891.70547.56 15345796

[B14] McPherson GC Campbell MK Elbourne DR Does using minimisation make a difference? empirical evidence from three multi-centre studies [abstract] Clin Trials 2012 9 456

[B15] Hewitt CE Torgerson DJ Is restricted randomisation necessary? BMJ 2006 332 1506 1508 10.1136/bmj.332.7556.1506 16793819 PMC1482349

[B16] McEntegart D Buyse M Achieving balance in clinical trials: an unbalanced view from EU regulators Applied Clin Trials 2004 13 36 40

[B17] Senn S Baseline Balance and Valid Statistical Analyses: Common Misunderstandings Applied Clin Trials 2005 14 24 27

[B18] Taves DR The use of minimization in clinical trials Contemp Clin Trials 2010 31 180 184 10.1016/j.cct.2009.12.005 20060500

[B19] ICH Steering Committee ICH Harmonised Tripartite Guideline E9; Statistical Principles for Clinical Trials Stat Med 1999 18 1905 1942 10532877

